# Molecular study of feline dermatophytosis and Toll‐like receptor 2 and 4 gene expression in their lesions

**DOI:** 10.1002/vms3.1120

**Published:** 2023-03-13

**Authors:** Anahita Kasmaei, Alireza Salimi, Javad Ashrafi Helan, Seyed Ali Shabestari Asl, Farzad Katiraee

**Affiliations:** ^1^ Department of Pathobiology Faculty of Veterinary Medicine, University of Tabriz Tabriz Iran; ^2^ Faculty of Veterinary Medicine Islamic Azad University of Tabriz Tabriz Iran

**Keywords:** cat, dermatophytosis, PCR, Toll‐like receptor

## Abstract

**Background:**

Pattern recognition receptors (PRRs) as the recognition of pathogenic fungal structures induce the secretion of cytokines by immune systems. Toll‐like receptors (TLRs) 2 and 4 are the main PRRs that recognize fungal components.

**Aim:**

The present study aimed to assess the presence of dermatophyte species in symptomatic cats in a region of Iran and to investigate the expression of TLR‐2 and 4 in cat lesions with dermatophytosis.

**Methods:**

A total of 105 cats suspected of dermatophytosis with skin lesions were examined. Samples were analysed by direct microscopy using potassium hydroxide (20%) and culture on Mycobiotic agar. Dermatophytes strains were confirmed by the polymerase chain reaction (PCR) amplification and then sequencing of the Internal Transcribed Spacer rDNA region. Also, for pathology and real‐time PCR studies, skin biopsies were taken by sterile single‐use biopsy punch from active ringworm lesions.

**Results:**

Dermatophytes were found in 41 felines. Based on the sequencing of all strains, *Microsporum canis* (80.48%, *p* < 0.05), *Microsporum gypseum* (17.07%) and *Trichophyton mentagrophytes* (2.43%) were the dermatophytes isolated from cultures. Cats under 1 year (78.04%) revealed a statistically significantly higher prevalence of infection (*p* < 0.05). Gene expression by real‐time PCR revealed the increased TLR‐2 and 4 mRNA levels in skin biopsies of cats with dermatophytosis.

**Conclusions:**

*M. canis* is the most prevalent dermatophyte species isolated from feline dermatophytosis lesions. Increased expression of TLR‐2 and TLR‐4 mRNAs in cat skin biopsies suggests that these receptors are involved in the immune response by recognizing dermatophytosis.

## Graphical abstract

Toll‐like Receptors (TLRs) 2 and 4 are the main PRRs that recognize fungal components. The present study aimed to investigate the expression of TLR‐2 and 4 in cat lesions with dermatophytosis. Increased expression of TLR‐2 and TLR‐4 mRNAs in cat skin biopsies suggests that these receptors are involved in the immune response by recognizing dermatophytosis.

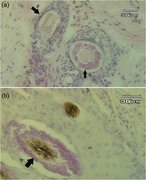

## 1 INTRODUCTION

Dermatophytosis is a common superficial infection in pets caused by a group of fungi, called dermatophytes, which belong to Ascomycota filamentous fungi. The growth of these fungi is typically limited to keratinized structures in the body (stratum corneum, hair, nail or claw). The dermatophytes are cosmopolitan fungi and include over 52 species from the genera, Arthroderma, Nannizzia, Trichophyton, Microsporum, Epidermophyton, Lophophyton and Paraphyton (de Hoog et al., 2017). The most prevalent dermatophyte species in small animals include *Microsporum canis* and *Trichophyton mentagrophytes. M. canis* is the most commonly identified dermatophyte, accounting for over 80% of dermatophytosis in dogs and cats, while *T. mentagrophytes* infection is uncommon (5%), and *M. gypseum* infection is rare (1−3%) (Katiraee et al., 2021; Paryuni et al., 2020).

The clinical presentation and disease progression of dermatophyte infection depends on several factors, such as the topography of infection, sex, age, the host's immune status and the species infecting dermatophytes (Cambier et al., 2016; Jackson & Marsella, 2012; Oliveira et al., 2015). Despite much progress in research into the immune response in human dermatophytosis, little is known about the host's immune response to dermatophytosis in animals. Interaction between the fungal component and the host immune system initiates a significant of innate immune responses, but further studies are needed to investigate the role of immune responses in dermatophyte infection.

Fungal pathogens are identified by specific receptors on immune system cells that are called pattern recognition receptors (PRRs) and are expressed on immune cells, including keratinocytes (Mignon et al., 2008). The recognition of pathogenic fungal structures by PRRs causes the secretion of pro‐inflammatory cytokines by immune system cells (Vermout et al., 2008). If these mechanisms are not sufficient to prevent fungal invasion, the adaptive immune system is turned on (Celestrino et al., 2019; Celestrino et al., 2021).

Toll‐like receptors (TLRs) are a family of PRRs as crucial signalling receptors mediating innate immune recognition, comprising 10 receptors with distinct recognition profiles. Keratinocytes are known to express TLRs‐1 to 6, as well as TLR‐9 (Oliveira et al., 2015).

After the ability of TLRs to control fungal infections in Drosophila was described, further research showed that they mediate the host's response to microbial pathogens. (Lemaitre et al., 1996). To stimulate an inflammatory response, TLR intracellular signalling is mediated by TIR domain‐containing adapter‐inducer interferon‐b (TRIF) and myeloid differentiation primary response 88 (MyD88). Fungal ligands that bind to TLR are not entirely determined. Anyway, an experimental study showed that TLRs cross‐signal together with C type lectins and modulate the antifungal defence (Burstein et al., 2020).

Also, TLRs act as a link between innate and acquired immune responses.

For example, cellular maturation and expression of stimulatory molecules, such as CD40 and CD80, occur when TLRs are activated on dendritic cells (DCs) by their respective pathogen‐associated molecular patterns (PAMPs). The activation of TLRs on DCs also results in an increased production of IL‐12, which subsequently promotes Th‐1 cell‐mediated adaptive immune response (Miller & Modlin, 2007). Besides, TLR‐2 is expressed in monocytes, macrophages and polymorphonuclear neutrophils (PMNs) and recognizes various ligands in the fungal cell wall (Celestrino et al., 2019). Keratinocytes and fibroblasts increase TLR‐2 and 4 mRNA expression on interaction with dermatophytes (Burstein et al., 2020). In this study, we aimed to investigate the expression of TLR‐2 and TLR‐4 in feline dermatophytosis lesions and clear the importance of these receptors in host immune response by the recognition of dermatophytes.

## 2 MATERIALS AND METHODS

### 2.1 Study population

Sampling was performed among cats suspected of having dermatophytosis that were referred to veterinary clinics in Tabriz, Iran, from September 2018 to December 2020. Thus, 105 cats in northwestern Iran were obtained and examined for evidence of dermatophytosis at the Mycology Lab of the Faculty of Veterinary Medicine, University of Tabriz. All demographic data were recorded and the results were analysed based on age, sex and hair length. Most of the infected cats were under 1‐year old and were kept indoors but sometimes went outside, as well.

The clinical manifestation of dermatophytosis is different. However, the best samples for dermatophyte testing will have the hair shaft or scales from areas of inflammation. Clinical samples (skin scrapings, scalp scales and hair) were collected in petri dishes and stored in plastic closed containers without humidity for transfer to the veterinary mycology laboratory of the Faculty of Veterinary Medicine, University of Tabriz. Also, skin biopsies were removed by a sterile, punch biopsy (size 3.5 mm), from the margins of ringworm lesions for pathology and real‐time PCR studies. Pathology samples were placed in formalin, and real‐time PCR samples were transferred inside RNA later solution.

### 2.2 Direct examination

Routinely, all clinical samples were pre‐digested and examined with potassium hydroxide (20%) for hyphae and arthroconidia by microscopy examination.

### 2.3 Fungal culture and identification

All samples were inoculated into Sabouraud dextrose agar containing chloramphenicol (50 mg/L) and mycobiotic agar. The inoculated agar media were incubated at room temperature for 4 weeks and checked daily. Each grown dermatophyte isolate on culture was identified based on its macroscopy and microscopy characteristics. The conidia were identified after lactophenol blue staining. Also, the Internal Transcribed Spacer (ITS)‐PCR method relying on the ITS1 and 2 and 5.8S ribosomal DNA subunit region was performed for the final diagnosis of dermatophyte species (Taghipour et al., 2021). Amplification was performed by the universal primers V9G (5′‐TTACgTCCCTgCCCTTTgTA‐3′) and LS266 (5′‐GCATTCCCAAACAACTCgACTC‐3′). The amplified ITS region was sequenced and a comparison of nucleotide sequences was carried out with reference fungal nucleotide sequences obtained from the Central Bureau of Fungal Cultures database at the Westerdijk Fungal Biodiversity Institute (http://www.cbs.knaw.nl/dermatophytes/BioloMICSID.aspx). The similarity of >99% to the reference ITS sequences was found.

### 2.4 Pathology

In this study, there are two study groups for pathology and gene expression: infected animal groups and the healthy control group. Skin tissue samples from the margins of lesions were collected and fixed in 10% buffered formalin, routinely processed and embedded in paraffin. Sections were cut and stained with haematoxylin and eosin.

### 2.5 RNA extraction

The RNA was extracted to evaluate the expression of TLR‐2 and 4 genes in dermatophyte isolates. The RNA extraction was performed using a Gen all nucleic acid extraction kit made in Korea. The product was obtained based on the 260–280 nm absorption ratio of about 1.2–2 in a spectrophotometer. This method was used to extract RNA from all strains. The extraction steps are summarized as follows:

Approximately 5 g of the tissue sample was placed in a microtube, and 750 μL of RL buffer was added and placed at room temperature for 10 min. Then, 150 μL of chloroform solution was added to the microtube and vortexed for 15 s. It was then placed at real‐time (RT) for 3 min. The tube was centrifuged at 13,000 rpm for 12 min at 4°C. After centrifugation, the solution assumed a three‐phase state. Then, 400 μL of the supernatant was poured into other tube, and 400 μL of ethanol was added. The resulting solution was poured into a filter and placed in a tube. The filter was centrifuged for 1 min at 13,000 rpm. Next, 700 μL of purified water solution was poured into a filter and centrifuged at 13,000 rpm for 1 min. The filter was centrifuged separately for 2 min at 13,000 rpm. Next, 50 μL of diethyl pyrocarbonate (DEPC)‐treated water solution was poured in the middle of the filter and placed at room temperature for 3 min. The filter was centrifuged for 13 min at 13,000 rpm to yield the final product, RNA. After RNA extraction, its quality was evaluated by agarose gel electrophoresis and staining with Safe Stain®.

### 2.6 cDNA synthesis

After disclosing the quality and concentration of RNA, using the cDNA synthesis kit of Yektatahiz Azma Company, cDNAs were made from RNAs according to the kit instructions and then stored at −20°C. The protocol introduced by the company was used to generate single‐stranded cDNA using real‐time PCR. All reaction compounds were centrifuged for several seconds after thawing and kept on ice. The following compounds were added to a 0.2 sterile free nuclease nucleotide on ice: 0.1 ng∼5 μg Template RNA, 1 μL Random Hexamer, DEPC‐treated water up to 13.4 μL. After gently mixing, the mixture was incubated for 5 min at 70°C and then cooled on ice. The cDNA synthesis mix, which contained 4 μL 5x first‐strand buffer, 1 μL dNTPs (10 mM each), 0.5 μL RNasin (40 U/μL) and 1 μL M‐MLV (200 U/μL), was added to the previous reaction product to bring the total volume of the reaction product to 20 μL. The resulting reaction mixture was incubated at 37°C for 1 h and then at 70°C for 5 min and, finally, transferred to a −20°C freezer.

### 2.7 Real‐time PCR

The real‐time PCR method was used to examine part of the exon of the TLR‐2 and 4 genes, as well as part of the ACTIN gene in the RNA extracted from the samples, and to determine the expression level of TLR‐2 and 4 genes in comparison with the reference gene. Amplification was performed in a reaction mixture with a total volume of 20 μL using the master mix (2X real‐time PCR Master Mix), SYBR Green, a ready‐to‐use solution for quantitative real‐time PCR. The real‐time PCR was carried out with a Stratagene Mx3000p system.

Melting curve analysis was performed to confirm the amplification of a single product in each reaction. In this study, relative real‐time PCR data were studied using the 2^−ΔΔCt^ method. The relative level of each mRNA in the sample was calculated and expressed as a ratio relative to the endogenous ACTIN housekeeper locus. The nucleotides primer used are shown in Table 1.

**TABLE 1:** Primers used in real‐time PCR

Gene	Nucleotide sequences of three‐pair primers used in real‐time (RT)‐PCR
ACTIN	F: CACTGGTATTGTCATGGACTCT R: GTAGCACAGCTTCTCCTTGAT
TLR2	F: GAAAGTTATCGTCGTGGTGTC R: CTGCTGAGAAACTGCCAAGT
TLR4	F: TGCTTCAGGGTTTCATCCA R: GACACTCGCTCAGCTTCTTG

### 2.8 Statistical analysis

The data were pooled and analysed using GraphPad Prism 9.0.2 statistical software. The results are provided as the mean and standard errors of the mean. A *t* test was used to make the two‐group comparisons. The chi‐square (*X* ^2^) test was applied to assess statistical differences between the test groups. The level of significance was set at *p* < 0.05.

## 3 RESULTS

### 3.1 Clinical observation study

In clinical observation using Wood's lamp, acute fungal lesions were observed in the ears, forehead, nose, snout, under the chin, hands, nails, under the abdomen, behind the waist and at the base of the tail, which were often round with hair loss and more severe inflammation in the margins. There was also seborrhoea in these areas, sometimes due to the itching of bloody scabs. In cats whose hands were infected, snouts were also seen due to the licking of these areas. In a small number of cases, the size of the lesions was over 5 cm, and the wounds were usually not deep, but there was no symmetrical shape. In infected animals, the outer covering was usually opaque and dark, and hair dampness and ringworm hair loss were also visible. Chronic lesions showed less inflammation and redness.

### 3.2 Direct microscopy and culture study

**FIGURE 1:**
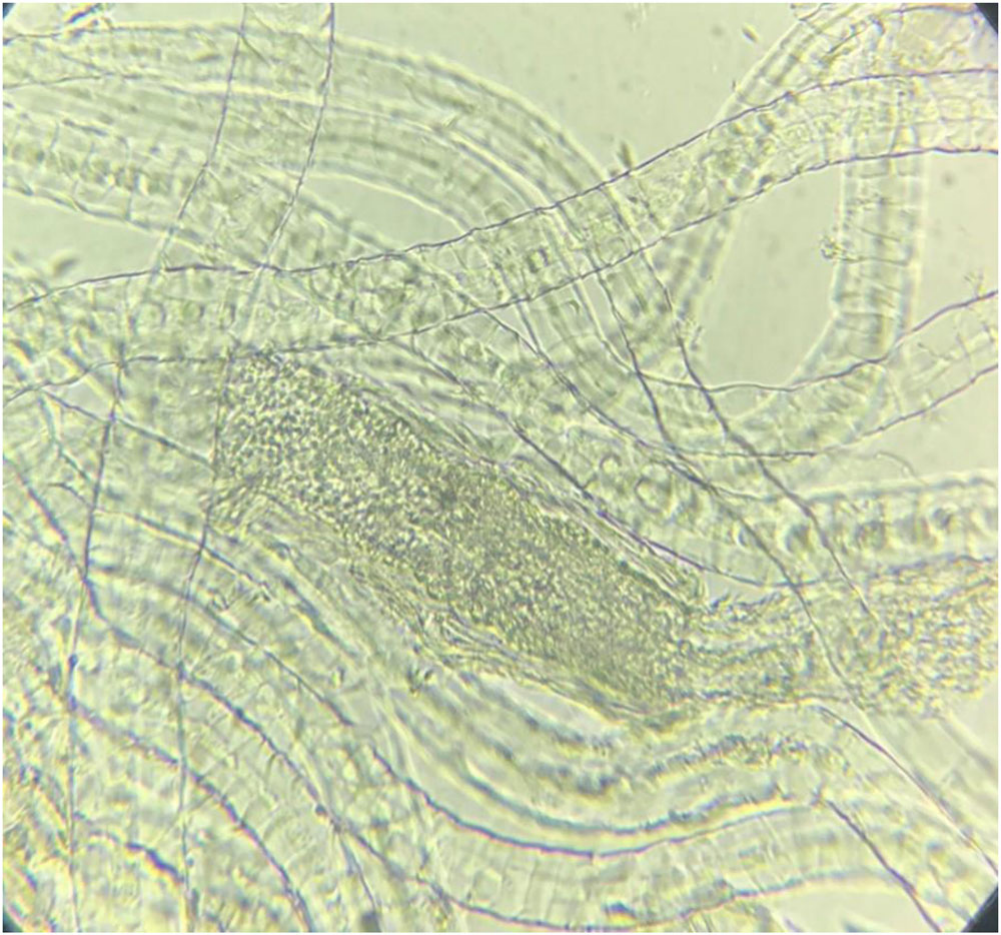
Direct microscopic examination (KOH 20%) of cat hair, arthrospores were seen in the hair shaft.

Out of 105 samples studied, 40 samples were positive by direct microscopic examination and had fungal elements, including mycelium or arthrospores (Figure 1). Table 2 shows the results of the direct microscopic examination in cats suspected of dermatophytosis. In this study, the rate of positive results was higher in long hair cats, which included 73.13% of infected cats. Also, in terms of gender, the number of females was slightly more than that of males (56.09%).

**TABLE 2:** Direct microscopic examinations and fungal cultures

		Cats	
Test results		Number	Percentage (%)
Microscopy	Positive	40	38.09%
	Negative	65	61.90%
Culture	Positive	41	39.04%
	Negative	64	60.95%

From an age perspective, dermatophytosis was seen in most animal age groups, but the disease was significantly more common in young animals. The results of direct microscopic examination, which indicated dermatophytosis infection, showed that 78.04% of infected cats in the sample were under 1‐year‐old at the time of infection, and 21.95% were over 1‐year old (*p* < 0.05) (Table 3). In other words, the development of dermatophytosis was associated with young age.

**TABLE 3:** Demographic data of cats with suspected cases of dermatophytosis

		Cats	Percentage (%)
Age	<1‐year old	32	78.04%
	>1‐year old	9	21.95%
Sex	Female	23	56.09%
	Male	18	43.90%
Habitat	Indoor	19	46.34%
	Outdoor	22	53.65%
Disease in owner	Yes	15	36.58%
	No	26	63.41%
Outer covering	Long‐hair	30	73.13
	Short‐hair	11	26.82

Fungal culture experiments were performed on Sabouraud dextrose agar containing chloramphenicol (SC) and Sabouraud dextrose agar containing chloramphenicol and cyclohexime (SCC) media. A total of 41 culture samples were dermatophyte‐positive (39.04%). One (0.95%) sample which was negative in microscopy examination, dermatophytes grew in a culture medium (Table 2). In the culture experiment, the characteristics of the grown colonies were examined. The positive culture of the grown colonies was consistent with the typical colony of *M. canis*. Some colonies also showed an atypical appearance and did not have any colour or pigment. The amount of sporulation was different in different types of colonies. In 30% of the cases, colonies without macroconidia were observed, for which macroscopic and microscopic diagnoses are difficult and depend on molecular diagnosis.

Based on the sequencing of all strains, *M. canis* (80.48%), *M. gypseum* (17.07%) and *T. mentagrophytes* (2.43%) were the dermatophytes isolated from cultures. There were no significant differences between the obtained sequences and reference ITS sequences.

### 3.3 Pathological study

**FIGURE 2:**
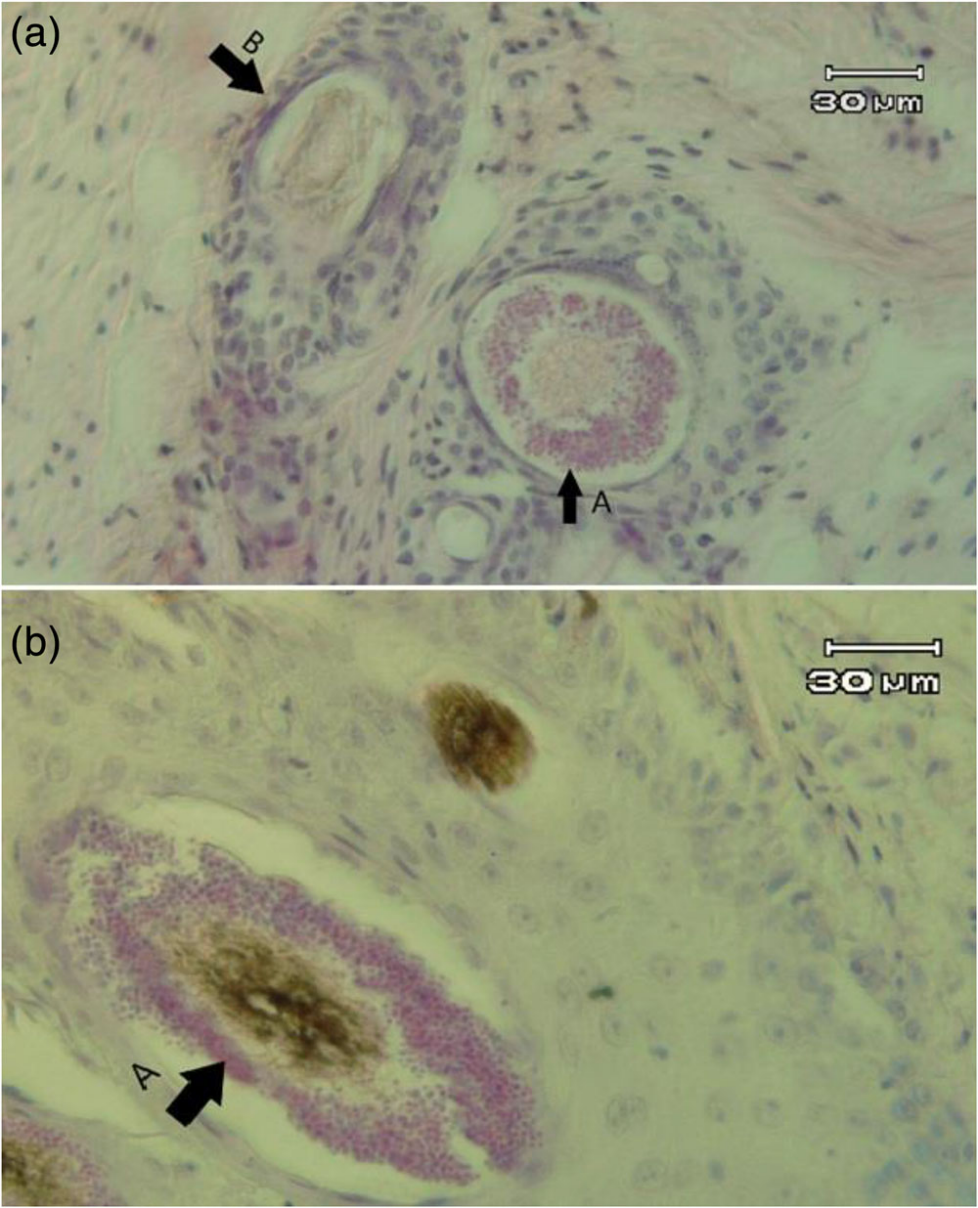
Magnification 400, severe infection of hair follicle in the form of ectothrix (a), normal hair follicle (b).

In this study, skin punches were taken from the margins of dermatophytosis lesions. After fixation in formalin, a pathology slide was prepared and studied under a light microscope at different magnifications. Infection of hair follicles with *M. canis* could be seen in the forms of endothrix, ectothrix and favus. The frequency of the ectothrix (73.17%) (Figure 2) form was higher than the endothrix (7.31%) and favus (19.51%).

### 3.4 Real‐time PCR study and TLR 2 and 4 gene expression

**FIGURE 3:**
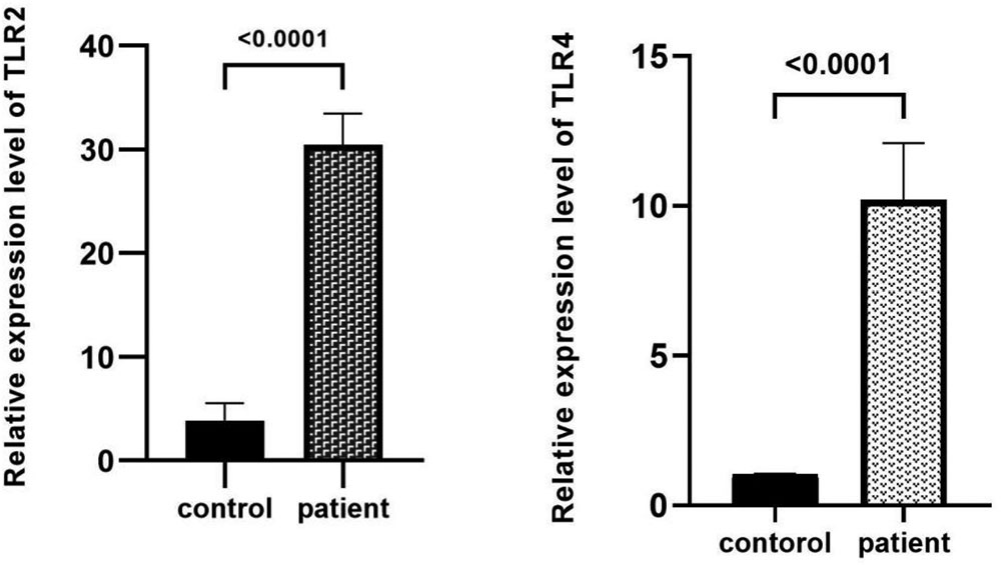
The bar chart shows the relative expression of TLR2 and 4 in control and patients. In patients, the expression of the TLR2 and 4 is increased <0.0001.

The RNA extraction, cDNA conversion and real‐time PCR were performed on 33 skin biopsies infected with dermatophytosis and control samples. The expression of TLR‐2 and 4 genes was higher in all samples than in controls. Also, the pure value of the *t* test to compare between healthy control and infected groups was less than 0.005, a significant change was confirmed in gene expression (Figure 3).

## 4 DISCUSSION

Dermatophytosis is a zoonosis fungal infection. Thus, animal contact is an effective factor that causes the transmission of dermatophytes infection. Dermatophytosis occurs at all ages but frequently is seen in cats less than 12 months of age. This is in line with our results, as we observed that 78.04% of infected cats were less than 1 year of age. In agreement with our results, there are similar studies conducted by Lefkaditis (2006), Cafarchia et al. (2013), Gangil et al. (2012) and Shokri and Khosravi (2016). The current article revealed that in 41 dermatophytosis cases, there were 56.09% females and 43.90% males; but, there were no significant differences in dermatophytosis in males and females. Other studies have reported that male cats are more likely to be affected by dermatophytosis (Cafarchia et al., 2004), although most other reports have found no association between sex and the presence of dermatophytosis (Cafarchia et al., 2013; Debnath et al., 2016). Identification of the causative agent of dermatophytosis is a key step in fungal epidemiological studies. Because of the resemblance between dermatophytes, different physiological and phenotypic methods are essential for accurate identification. Due to the time‐consuming and insufficient of these methods to diagnosis, nowadays, the rDNA sequencing method is used for the identification of dermatophytes and the ITS region for the identification of dermatophyte species is exploited (Garg et al., 2009; Petrucelli et al., 2020).

In the present study, *M. canis*, *M. gypseum* and *T. mentagrophytes* were isolated from cats with dermatophytosis. The dermatophyte species identified in this study are similar to the species isolated from cats reported in Iran (Khosravi & Mahmoudi, 2003; Shokri & Khosravi, 2016) and other countries (Chermette et al., 2008). The most common genera of dermatophytes isolated from our samples were *M. canis*, which is in agreement with earlier studies (Beigh et al., 2014; da Costa et al., 2013; Seker & Dogan, 2011). However, some studies contradict our results (Sarifakioglu et al., 2007). Possibly, the use of molecular techniques for dermatophyte identification is the reason for the discrepancy between studies.

Dermatophytosis is a cutaneous fungal disease, which is difficult to treat and usually chronic. Previous studies have shown that TLRs play critical roles during contracting infectious fungal diseases (Celestrino et al., 2019). This study showed that mRNAs expression of TLR 2 and 4 increased in cat skin, suggesting that these immune proteins played a role in the host immune response through the recognition of dermatophytes. It is the first time that feline skin biopsies are clinically examined for TLR‐2 and TLR‐4 gene expression in animal dermatophytosis. Other studies showed the importance of these receptors in the host immune response against fungal infection. In human neutrophils, the TLR‐2 and TLR‐4 are involved in recognizing fungal PAMPs and, consequently, help to create a protective immune response (Acorci‐Valério et al., 2010). Another study showed that cat PMNs secreted pro‐inflammatory cytokines, including IL‐8, TNFa and IL‐1b, in response to *M. canis* arthroconidia stimulation (Cambier et al., 2016). On the other hand, TLR‐2 and TLR‐4 expression increased in cats PMNs exposed to live and heat‐killed arthroconidia from *M. canis*; these results strongly suggest the role of these receptors in immunity against this dermatophyte (Cambier et al., 2016).

In comparison, Oliveira et al. (2015) described that the expression of TLR‐4 (but not of TLR‐2) was decreased in lesions of patients with disseminated dermatophytosis, which indicates the possible mechanism for the chronicity of dermatophytosis. Likewise, in other mycology studies, we could find the importance of these receptors. The study results on *Trichophyton rubrum* demonstrated that TLR‐2 is important for the elimination of *T. rubrum* conidia and the production of pro‐inflammatory cytokines by human monocytes (Celestrino et al., 2019). The measurement of TLR‐2, 4 and dectin‐1 mRNA in the HaCaT, co‐cultured with the arthroconidia of *T. rubrum*, showed that TLR‐2, 4 and dectin‐1 mRNA expression increased in response to *T. rubrum* conidia (Li et al., 2011). Also, dectin‐1 recognizes b‐glucans, whereas TLR‐2 and TLR‐4 are receptors that bind to the mannans of fungi. Mannans are more external components than b‐glucans in the cell wall of fungi. Therefore, mannan is more accessible to PRRs than b‐glucans (Brown, 2011). In addition, a study on *Aspergillus fumigatus* in China showed that the expression of TLR‐2 and TLR‐4 and the release of IL‐1β and IL‐6 increased in cells stimulated with supernatants or hyphae from *A. fumigatus*. The expression of pIκB was also enhanced after exposure to the supernatant and hyphae. This finding suggests that the increased expression of TLR‐2 and TLR‐4 in response to *A. fumigatus* may result in the cytokines expression, which activates underlying stromal keratocytes, and recruits polymorph nuclear neutrophils to the infection site (Zhao & Wu, 2008). Additionally, a study on *Candida albicans* indicated that TLR‐2 is involved in macrophage‐mediated anticandidal activity, while the secretory response to *C. albicans* depends on TLR‐4 (Blasi et al., 2005). In Brasch's study on pityriasis versicolor, the expressions of TLR‐2 and TLR‐4 were more pronounced in infected skin than in normal skin (Brasch et al., 2014). All of these studies showed that the expression of TLR‐2, TLR‐4 and cytokines in response to different fungal species strongly depends on the host's genetic base and the type of fungal pathogen, indicating that these receptors have remarkable roles in the antifungal response.

We concluded from our findings that TLR‐2 and TLR‐4 are involved in recognizing *M. canis*. These receptors could also induce the secretion of pro‐inflammatory cytokines and lead the infection to heal. In this way, these receptors inhibited the preservation of dermatophytosis and stopped chronicity. However, not much has been discovered about these mechanisms in innate immunity. The characterization of these receptors would help understand the mechanisms of immunity against *M. canis*. In future studies, it would be beneficial to determine the expression of pro‐inflammatory cytokines of TLR‐2, TLR‐4 and in felines with chronic dermatophytosis lesions. Despite the role of PRRs to be investigated in human superficial dermatophytosis, they have rarely been studied in pets.

## AUTHOR CONTRIBUTIONS

Anahita Kasmaei: Data curation, investigation, writing—original draft. Alireza Salimi: Data curation, investigation, methodology. Farzad Katiraee: Conceptualization, methodology, supervision, validation, writing—review & editing. Javad Ashrafi Helan: Investigation, supervision, writing—review & editing. Seyed Ali Shabestari Asl: Investigation, methodology.

## CONFLICT OF INTEREST STATEMENT

The authors declare that there is no conflict of interest regarding the authorship and publication of this research article.

## ETHICS STATEMENT

The authors confirm that the ethical policies of the journal, as noted on the journal's author guidelines page, have been adhered to.

### PEER REVIEW

The peer review history for this article is available at https://publons.com/publon/10.1002/vms3.1120.

## Data Availability

Data are openly available in a public repository that issues datasets with DOIs.
